# Complicated appendicitis presenting as anterior abdominal wall abscess in a diabetic patient: A case report

**DOI:** 10.1016/j.ijscr.2025.111390

**Published:** 2025-04-30

**Authors:** Samrat Shrestha, Mecklina Shrestha, Kaushal S. Thapa, Ritesh Raj Pandey

**Affiliations:** a National Academy of Medical Sciences, NAMS, Bir Hospital, Department of General Surgery, Kathmandu, Province-3, Nepal; b College of Medical Sciences(CoMS), Department of Emergency Medicine, Bharatpur, Kathmandu, Province-3, Nepal

**Keywords:** Complicated appendicitis, Abdominal wall abscess, Diabetes, Perforated appendix, Laparotomy, Case report

## Abstract

**Introduction:**

Acute appendicitis is one of the most common surgical emergencies (8.6 % in men and 6.4 % in women), with varying presentations, including complications such as appendicular abscess. While the typical clinical course involves right lower quadrant pain and fever, rare complications can present with atypical symptoms, particularly in high-risk patients such as those with diabetes.

**Presentation of case:**

A 55-year-old diabetic female presented with a 10-day history of worsening abdominal pain, foul-smelling discharge at the anterior abdominal wall below the umbilicus, and fever. Imaging revealed an appendicular abscess extending into the anterior abdominal wall. Emergency exploratory laparotomy showed a perforated appendix with a purulent collection in the peritoneal cavity extending to the anterior abdominal wall, requiring drainage and right hemicolectomy.

**Discussion:**

Perforated appendicitis (incidence of 20–30 %), particularly in diabetic patients, can lead to localized abscesses or soft tissue infections in atypical locations such as the abdominal wall. These rare complications are more likely in immunocompromised individuals, including those with diabetes, where the incidence of perforated appendicitis is notably higher. Early imaging with ultrasonography or Contrast Enhanced Computed Tomography is critical for identifying complicated appendicitis and guiding surgical intervention.

**Conclusion:**

Anterior abdominal wall abscesses as a complication of perforated appendicitis are rare but significant. This case underscores the importance of early and accurate diagnosis, supported by imaging, to guide appropriate surgical management. Timely intervention can help prevent life-threatening conditions such as necrotizing fasciitis, improve patient outcomes, and reduce the risk of postoperative complications, particularly in high-risk populations.

## Highlights

• Acute appendicitis is one of the common surgical cases with lifetime incidences of 8.6% of men and 6.4% of women.
• Anterior abdominal wall abscess due to perforated appendicitis is rare; common in diabetics and with impaired immunity.
• Appendicular perforation is more common in diabetics (38.9%) than non-diabetics (18.5%), showing higher complication risk.
• Ultrasonography and Contrast-enhanced computed tomography are crucial for diagnosis and guiding the surgical approach.
• Early diagnosis and timely surgical intervention are crucial to prevent severe complications, improving patient outcomes.

## 1 Introduction

Acute appendicitis is one of the common surgical cases that are encountered in surgical practice with lifetime incidences of 8.6 % of men and 6.4 % of women [1]. Most cases of acute appendicitis are diagnosed based on classical history and clinical examinations, can be confirmed by radiological investigations, and are managed surgically, but appendicitis can also be managed with non-operative management [2,3]. Complicated appendicitis has various presentations; it can result in a spectrum of complications, including phlegmon, intra-abdominal abscess, generalized peritonitis, appendicocutaneous fistula, and sepsis [3]. Early diagnosis and treatment are crucial to avoid morbidity and mortality. Soft tissue infections present as swelling, pain, and erythema, and appendicular abscesses presenting as abdominal wall abscesses are very rare [4,5]. Morbidity in complicated appendicitis ranges from 20 % to 30 %, often due to postoperative complications such as surgical site infections, intra-abdominal abscesses, and prolonged ileus [3,5,6]. The mortality rate for complicated appendicitis is generally low in healthy adults but can increase to 1–4 % (in low and middle-income countries) in elderly or immunocompromised patients, including those with diabetes mellitus, due to delayed presentation, atypical symptoms, and impaired host defenses [6,7]. This case describes a rare complication of perforated appendicitis, which presents as an anterior abdominal wall abscess and its course in our hospital. This case has been reported according to the revised SCARE guidelines 2023 [8].

## 2 Case report

A 55-year-old female with type 2 diabetes presented to the emergency department with a 10-day history of progressively worsening lower abdominal pain, associated with foul-smelling purulent discharge from the anterior abdominal wall below the umbilicus and fever. However, there was no history of nausea, vomiting, melena, or per-rectal bleeding. The patient had normal bowel and bladder habits. She had a history of total abdominal hysterectomy with bilateral salpingo-oophorectomy and omentectomy for a complex ovarian cyst 4 years prior. There was swelling over the previous incision site. The patient had been managed with oral hypoglycemic therapy, specifically Metformin 500 mg twice daily, and had no prior history of insulin use. The patient denied any previous diabetic complications.

**Fig. 1:**
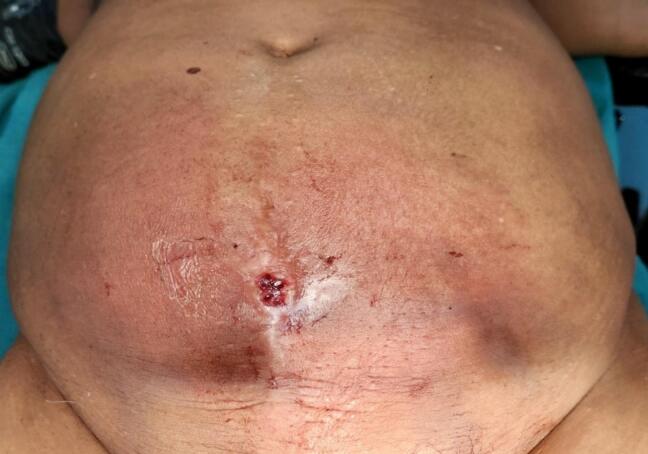
Indurated erythematous swelling in hypogastrium with an ulcerative lesion at the center.

On examination, the patient's vitals were stable. A Pfannenstiel incision was present at the lower abdomen. Approximately 10 cm × 12 cm indurated swelling was present over the hypogastrium, which was tender and erythematous. There was a 1 cm × 1 cm ulcer - 12 cm caudal to the umbilicus at midline with an actively draining, foul-smelling purulent discharge from the anterior abdominal wall (Fig. 1). Tenderness was noted over the swelling; however, the rest of the abdomen was nontender, there was no, guarding, or rigidity.

**Fig. 2:**
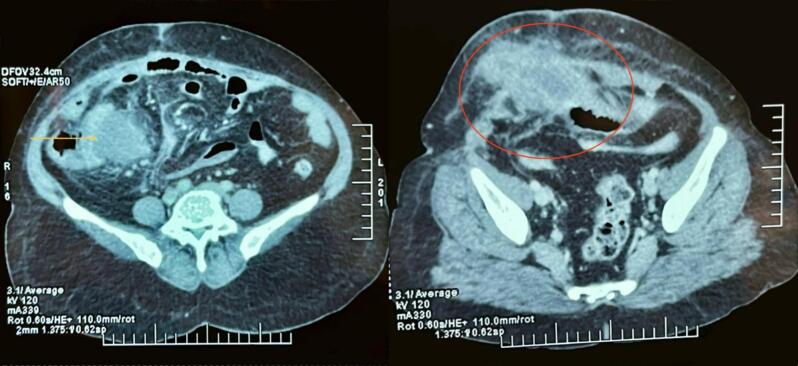
CECT of abdomen and pelvis axial view shows a peripherally enhancing lesion with a thick, irregular wall and a central collection in the right lower abdomen (yellow arrow), extending into the right anterior abdominal wall (red circle). CECT: Contrast-Enhanced Computed Tomography. (For interpretation of the references to colour in this figure legend, the reader is referred to the web version of this article.)

**Fig. 3:**
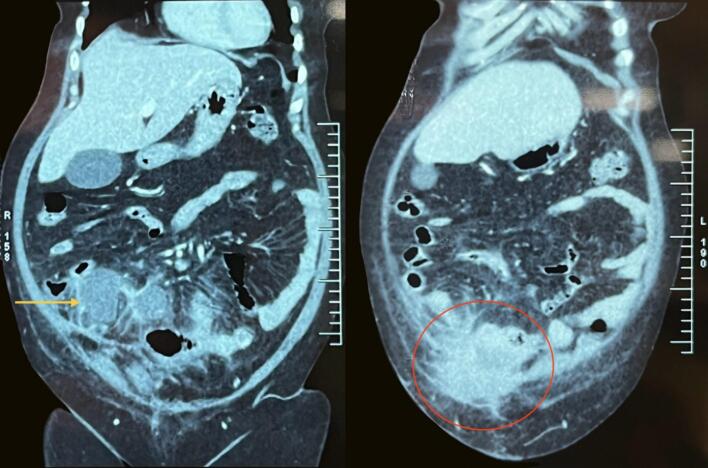
CECT abdomen and pelvis coronal section showing hyperechoic lesion in the right lower quadrant of the abdomen (yellow arrow) extending into the anterior abdominal wall (red circle). CECT: Contrast-Enhanced Computed Tomography. (For interpretation of the references to colour in this figure legend, the reader is referred to the web version of this article.)

The patient's white blood cell count was elevated at 17,500 cells/μL (Normal level: 4000–11,000/μL), random blood glucose level was 9.9 mmol/L (Normal level: 4.4–7.8 mmol/L) and C-reactive protein (CRP) was 249.1 mg/L (Normal range: 0.3 to 1.0 mg/L). The rest of the laboratory parameters were within normal limits. Ultrasonography (USG) of the abdomen and pelvis showed a heterogeneously hypoechoic collection measuring approximately 10 cm × 4 cm × 8 cm (estimated volume ∼ 210 cc) at the right lower abdomen, extending from the subcutaneous plane to the intramuscular plane. There was a fascial defect measuring 2.1 cm at the previous incisional site. Contrast-enhanced computed tomography (CECT) revealed a peripherally enhancing lesion with a thick, irregular wall and central collection of approximately 9 cm × 5 cm × 9 cm in the right lower abdomen, extending into the right anterior abdominal wall, suggestive of an appendicular lump with abscess (Figs. 2 and 3). The patient was diagnosed with an anterior abdominal wall abscess resulting from an appendicular lump with abscess formation.

**Fig. 4:**
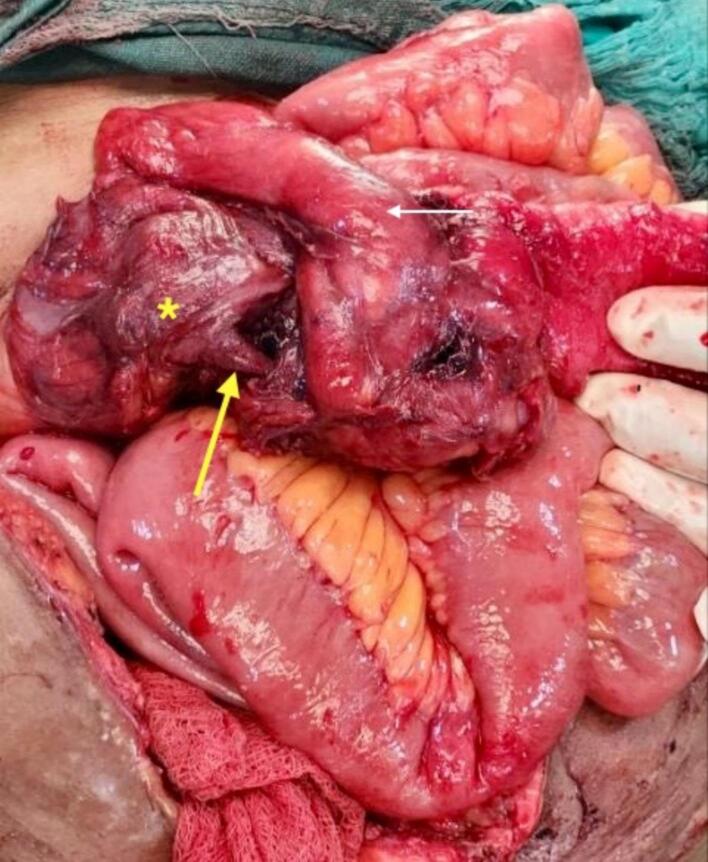
Intraoperative picture showing a lump formed by the cecum (asterisk), appendix (yellow arrow), ileum (white arrow), and omentum. (For interpretation of the references to colour in this figure legend, the reader is referred to the web version of this article.)

An emergency exploratory laparotomy was scheduled. Intraoperatively, a 40 ml purulent collection was found in an abscess cavity, at the right lower anterior abdominal wall, which communicated with the peritoneal cavity through a 2 cm × 2 cm fascial defect. The appendicular lump was formed by the cecum, appendix, ileum, and omentum (Fig. 4), with a 150 ml purulent collection in the peritoneal cavity, draining into the skin through the anterior abdominal wall. An appendicular perforation was identified at the mid-shaft, complicated by thrombosis of the terminal branch of the ileocolic artery, leading to ischemic changes in the cecum. Intraoperatively, the thrombosis of the ileocolic artery was identified by lack of pulsation and discoloration of the involved bowel segment. Palpation revealed an absence of pulsatile flow in the ileocolic pedicle. The bowel segment supplied by the ileocolic artery appeared dusky, non-peristaltic, and non-bleeding on pinprick, consistent with ischemia. Drainage and debridement of the anterior abdominal wall abscess with drainage of the intraabdominal abscess, followed by a limited right hemicolectomy with an end-to-end ileocolic handsewn anastomosis was done. The abdominal drain was placed. The fascial defect was repaired primarily using non-absorbable interrupted sutures. The decision for a more extensive resection was guided by the patient's poorly controlled diabetes, increased risk of wound complications, and the need for definitive control of the septic focus. Debridement of devitalized tissue was also essential to minimize the risk of necrotizing soft tissue infection, a serious concern in diabetic patients.

Postoperatively, the patient was monitored in the ICU. Meticulous daily wound care with regular dressing changes and sterile technique was employed. The patient passed flatus on the 2nd postoperative day (POD), and bowel sounds returned before initiation of an oral diet. A liquid diet was started on the 3rd postoperative day (POD). Microbiological culture of the purulent discharge identified *Escherichia coli* and *Pseudomonas aeruginosa*. The antibiotics were titrated according to the culture and sensitivity report. A normal diet was initiated on the 5th POD. The abdominal drain was removed on the 7th POD, and the patient was eventually discharged on the 10th POD with normal postoperative recovery. 1 week later, during a routine follow-up in the outpatient department (OPD), the patient had no complaint with a clinically soft abdomen. The wound showed progressive healthy granulation, and subsequently secondary suturing was done. No signs of surgical site infection or wound complications were noted. There was no history of fever, chills/rigor, or abdominal pain after her discharge. USG confirmed complete resolution of the anterior abdominal wall and appendicular abscess. At 1 month, 3 months, and 6 months follow-up, the patient recovered completely with no signs of repeated infection.

## 3 Discussion

Acute appendicitis is the most common cause of acute abdomen presenting in emergency department admissions affecting all age groups, and about 25 % of all appendectomies are done for complicated appendicitis [3]. About 20–30 % of all cases have perforated appendicitis [5]. Perforated appendicitis can present as localized abscess or peritonitis but can also present as soft tissue infection of the anterior abdominal wall, groin, perineum, or lumbar region, with the most serious complication being necrotizing fasciitis (NF) [9,10]. Appendicular abscess presenting as anterior abdominal abscess is a very rare complication that is described in our case report. Soft tissue infections are frequently encountered in primary care. They typically affect the skin and subcutaneous tissue, with the lower extremities being the most common site. Patients usually present with pain, redness, and swelling of the affected area. Fever and systemic symptoms are generally mild [4]. In our patient, however, the presentation was unique due to the abdominal wall infection being secondary to a perforated appendix.

Risk factors for developing anterior abdominal wall abscess include diabetes, hematoma, and immunocompromised conditions [11]. The incidence of appendicular perforation in diabetic patients is 38.9 % compared to 18.5 % in non-diabetic patients [12]. In this case, the patient's type 2 diabetes mellitus may have contributed to the higher likelihood of perforation and abscess formation. This higher incidence is attributed to impaired immune response, delayed recognition of symptoms, and microvascular complications that lead to poor tissue perfusion and healing. Hyperglycemia in diabetic patients adversely affects neutrophil chemotaxis, phagocytosis, and intracellular killing of bacteria, increasing the risk of severe infections and delayed recovery [13]. Furthermore, diabetes-associated vascular changes reduce the effectiveness of inflammatory responses, allowing infections to progress more rapidly. Diabetic patients, due to chronic immune dysregulation, are predisposed to severe soft tissue infections, including NF, which can significantly worsen prognosis [14]. Infections in diabetic individuals are often polymicrobial, necessitating broad-spectrum antibiotic coverage [15]. The most common organisms responsible for these infections are *Escherichia coli*, *Klebsiella pneumoniae*, Streptococcus spp., and Enterococcus spp., followed by *Pseudomonas aeruginosa* [16]. Poor glycemic control has been linked to increased bacterial load and resistance, making targeted antibiotic therapy crucial in such cases [15].

Gockel et al. reported a rare case of an appendicocutaneous fistula resulting from appendix perforation with the formation of a perityphilitic abscess. Drawing a parallel to empyema necessitans, which penetrates the chest wall, they referred to this condition as appendicitis necessitatis [17]. Bulus et al. also reported an appendicocutaneous fistula as a very rare complication of acute appendicitis [18]. Additionally, Mohamed et al. described a geriatric patient who presented with an anterior abdominal wall abscess, characterizing it as an atypical manifestation of appendicitis. They noted that diagnosing appendicitis in geriatric patients is challenging, with a high risk of perforation leading to prolonged surgery and increased morbidity and mortality [19]. This patient's prior hysterectomy surgery site became the locus for the abscess, which further complicates the typical clinical presentation of appendicitis. Diagnosis of abdominal abscess can be aided by radiological investigations like CT scan, USG, and magnetic resonance imaging (MRI), which can also show the site and source of the abscess [3,20]. It is very important to thoroughly investigate cases of anterior abdominal wall abscesses because anterior abdominal wall abscesses commonly, but not exclusively, arise from intra-abdominal causes [21].

Drainage of the abscess with debridement of the necrotic skin under general anesthesia with appendectomy is the treatment option in such cases [22,23]. Diabetic patients undergoing surgery are at an increased risk of postoperative complications, including surgical site infections, delayed wound healing, and anastomotic leakage, necessitating close monitoring and aggressive glycemic control [24]. Studies have indicated that perioperative hyperglycemia correlates with increased mortality and longer hospital stays in such patients [25].

This comprehensive surgical approach led to a positive postoperative recovery with complete resolution of symptoms. Imaging and surgical intervention are necessary for patients to prevent morbidity and mortality, and this patient has a high chance of developing surgical site infection. Nevertheless, final confirmation of diagnosis can be achieved during surgical exploration with histological and microbiological workups [4,26]. While several studies report abscess formation in the abdominal cavity, our case is unique due to the presentation of an abdominal wall abscess, which is rarely seen as a complication of appendicitis. Previous case reports mention appendicitis-related abdominal wall abscess, but the extent and severity of the infection in our case required surgical debridement of the anterior abdominal wall, something not frequently encountered in other cases [17,23]. A notable aspect of our case is the ileocolic artery thrombosis identified during surgery, leading to ischemic bowel changes. This is a rare complication of appendicitis, as discussed by Kumar et al. and Dsouza et al. [10,22]

## 4 Conclusion

This case presents a rare complication of perforated appendicitis, where an anterior abdominal wall abscess developed in a diabetic patient. The unique aspect of this case lies in the atypical presentation of appendicitis as an abdominal wall abscess, requiring surgical intervention that included drainage, debridement, and a right hemicolectomy. Diabetic patients are at an increased risk of severe infections due to impaired immune function, which contributed to the development of this unusual complication. Early diagnosis through imaging and prompt surgical intervention were key in preventing further morbidity, including life-threatening conditions like necrotizing fasciitis. This case underscores the importance of considering complex presentations in high-risk populations and highlights the role of imaging and early surgical management in achieving favorable outcomes.

## Author contribution

• Constructing hypothesis for the manuscript- Samrat Shrestha, Mecklina Shrestha.
• Planning methodology to reach the conclusion: Samrat Shrestha, Mecklina Shrestha.
• Organizing and supervising the course of the article and taking responsibility: Samrat Shrestha.
• Patient follow-up and reporting –Mecklina Shrestha, Ritesh Raj Pandey, Kaushal S. Thapa.
• Logical interpretation and presentation of the results- Samrat Shrestha, Mecklina Shrestha, Ritesh Raj Pandey, Kaushal S. Thapa.
• Construction of the whole or body of the manuscript- Samrat Shrestha, Mecklina Shrestha, Ritesh Raj Pandey, Kaushal S. Thapa.
• Reviewing the article before submission not only for spelling and grammar but also for its intellectual content- Samrat Shrestha, Mecklina Shrestha, Kaushal S. Thapa, Ritesh Raj Pandey.

## Consent

Written informed consent was obtained from the patient for publication of this case report and accompanying images. A copy of the written consent is available for review by the Editor-in-Chief of this journal on request.

## Ethical approval

Ethical approval is waived at our institution (National Academy of Medical Science, Bir Hospital) and this study was exempt from ethical approval at our institution, as this paper reports a single case that emerged during a normal surgical case report.

## Guarantor

Samrat Shrestha accepts full responsibility for the work and/or the conduct of the study, has access to the data, and controls the decision to publish.

## Research registration number

Not applicable.

## Funding

This research did not receive any specific grant from funding agencies in the public, commercial, or not-for-profit sectors.

## Conflict of interest statement

The authors have no conflict of interest to declare.
